# Tracing How the Emergence of Chronic Pain Affects Military Identity: A Narrative Inquiry of Pain Trajectories Among Canadian Veterans

**DOI:** 10.3390/healthcare13202655

**Published:** 2025-10-21

**Authors:** Umair Majid, Tom Hoppe, Phoebe Priest, Leane Lacroix, Nicholas Held, David Pedlar, Kerry Kuluski

**Affiliations:** 1 Institute of Health Policy, Management and Evaluation, University of Toronto, Toronto, ON M5S 1A1, Canada; 2 Canadian Institute for Military and Veteran Health Research [CIMVHR], Queen’s University, Kingston, ON K7L 3N6, Canada; nicholas.held@queensu.ca (N.H.);; 3 Faculty of Health Sciences, McMaster University, Hamilton, ON L8S 4L8, Canada; 4 Institute for Better Health, Trillium Health Partners, Mississauga, ON L5B 1B8, Canada

**Keywords:** chronic pain, military, veterans, identity, qualitative research, narrative inquiry

## Abstract

**Background/Objectives:** Military identity serves as a foundational lens through which service members navigate the events of everyday military and civilian life. However, the very process that cultivates a sense of unity and purpose can be a double-edged sword in civilian life. Although the prevalence and transition needs are known, few studies have explored how chronic pain specifically disrupts military identity in depth. This qualitative study explores three distinct trajectories through which Veterans with chronic pain experience identity change. **Methods:** This study used narrative inquiry involving two sets of in-depth interviews with 20 Veterans. Reflexive thematic analysis was employed to describe and differentiate three distinct trajectories of chronic pain. **Results**: Veterans with chronic pain experience identity change through three overlapping pain trajectories: (1) traumatic injury -> immediate discharge; (2) misdiagnosed/non-traumatic injury -> delayed discharge; and (3) cumulative wear and tear -> gradual discharge. Regardless of trajectory, chronic pain consistently disrupted military identity and forced Veterans to confront tensions between institutional expectations of stoicism and combat readiness and the physical realities of chronic pain during military service. Those interviewed described experiencing fragmented institutional support, uneven access to care, and the systemic invalidation of pain that did not conform to military ideals. **Conclusions**: These findings underscore the need for Veteran-centred approaches, including responsive services, comprehensive pain science education throughout military careers, early detection of conditions that can lead to chronic pain, and flexible care pathways tailored to the nuances of each pain trajectory and grounded in military culture and lifestyle.

## 1. Background

Identity is defined as “the distinguishing character or personality of an individual” [1]. For military service members, this concept serves as a foundational lens through which they navigate the battlefield and everyday life after service [2]. Enlistment often starts at a young age, where the military environment profoundly molds a sense of self. The structured, hierarchical nature of the military, with its strict training regimens and practices, instills a strong collective identity characterized by a mission-first approach, resilience, and discipline, which can sometimes overshadow personal health needs [3,4]. But the reason why identity is important for service members and Veterans is because it is deeply ingrained; it is forged through shared challenges and collective discipline with other service members under the purview of unlimited liability—a legal obligation which imposes unlimited cost to the service member including death—and continues to influence Veterans long after leaving service by coloring their perceptions of self-worth and belonging in a civilian world that often demands a different kind of identity [4].

Yet, the very rigor that cultivates such a powerful sense of unity and purpose in the military can be a double-edged sword in civilian life. The intense demands of military training do not merely build character; they can exact a physical and psychological toll that service members navigate for the remainder of their lives as Veterans [5]. Chronic pain—defined as persistent discomfort lasting more than three months [6,7]—has emerged for many Veterans as a frequent and insidious companion. After leaving service, more than 4 in 10 Veterans in the United States report experiencing chronic pain [8], with Veteran women reporting higher rates of chronic pain [9,10]. In Canada, an estimated 52% of Veterans report living with chronic pain [11]. Veteran women reported higher rates of chronic pain compared to men in 2014, but more recent data are needed [12], and the gender dynamics of chronic pain trajectories will be considered in the discussion section.

Although prevalence and transition needs are known, few studies have explored how chronic pain specifically disrupts military identity in qualitative depth. In this study, the term’ living experience’ refers to the experience of individuals currently navigating a situation, which reflects how their needs and meanings evolve as the situation unfolds. Research has shown that a strong understanding and awareness of how military culture can influence Veterans in civilian life can improve healthcare quality [13,14]. Moreover, a systematic review conducted on the transition needs of Veterans with chronic pain found identity as one of the most important challenges represented in the literature [15]. However, we believe that there is limited deep, qualitative, and exploratory research looking at military identity—how Veterans define themselves in the face of experiences and challenges from military service to civilian life. The systematic review on transition needs of Veterans with chronic pain found many other challenges that we believe intersect with or can be categorized as identity challenges, such as finding purpose and meaning, role conflict, relationships, and employment [15]. This ambiguity of identity and related concepts and ideas necessitates a deep exploration of identity and identity challenges from the perspective of Veterans and how it can intersect with chronic pain to leave an enduring impact on their lives.

### 1.1. Military Culture, Mindset and Identity

**Figure 1:**
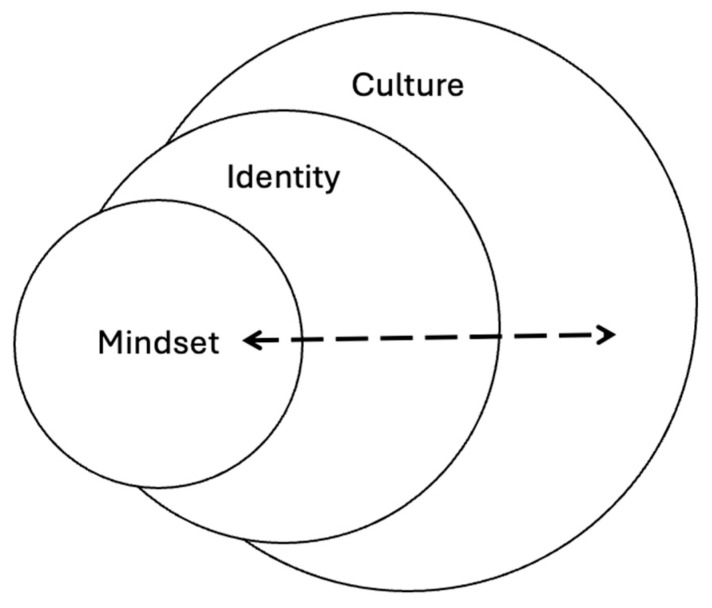
Visual depiction of culture, identity and mindset.

Military culture, identity, and mindset are interconnected yet analytically distinct constructs that shape the experience of military personnel. Military culture refers to the shared institutional values, norms, symbols, and practices that govern military life and socialization, emphasizing hierarchy, discipline, and emotional control [16]. It is through this military culture that military identity is internalized, which is how Veterans describe themselves during military service and civilian life. Military identity is shaped by service experiences and role integration and is often characterized by loyalty, cohesion, and a persistent “soldier self,” even post-discharge [17,18]. Military mindset represents the cognitive and emotional orientation developed through cultural and identity formation, influencing how service members interpret missions, respond to threats, and make decisions under pressure [19,20]. While culture provides the external structure and mindset informs action, identity operates as the lived and living bridge between the two that influences transitions, adaptability, and reintegration beyond active service [17]. Figure 1 shows the connection between these three concepts visually.

### 1.2. Conceptual Framework: Identity Change

Existing research on identity transformation suggests that shifts in self-concept can occur gradually and abruptly across diverse populations [21,22,23,24,25]. For example, subtle internal conflicts and value dissonance have prompted incremental changes in identity, as evidenced by studies examining health-adverse behaviors, such as smoking [22]. Conversely, traumatic events may catalyze rapid and profound reconfigurations of self-perception, leaving individuals struggling to reconcile their past identities with their emergent self-understandings in evolving contexts [26,27]. Such abrupt transitions are often associated with diminished self-esteem, pervasive identity confusion, and challenges in maintaining stable interpersonal relationships [28]. Such insights not only illuminate the mechanics of identity change but also underscore the necessity of tracing how chronic pain emerges and exerts its influence across the transition from military to civilian life.

For Veterans, this conceptual lens becomes particularly salient. The disciplined, resilient identity cultivated through rigorous military training is intrinsically linked to a narrative of strength and invulnerability. Yet, the emergence of chronic pain—often rooted in service-related injuries—introduces a disruptive element that challenges this established self-image. As physical and psychological suffering materializes, the once-cherished ideals of discipline and resilience are undermined, compelling Veterans to confront a transformed identity that diverges markedly from their military self-conception [29]. In this way, chronic pain is not merely a physical ailment but a catalyst that reshapes identity and potentially erodes the narrative of stoicism and strength that has long defined their lives.

This narrative study explores three distinct trajectories through which Veterans with chronic pain experience identity change. By tracing the emergence of chronic pain from its origins in military service to its enduring influence on identity in civilian life, this study explores the nuanced ways identity changes amid sustained physical and emotional distress.

## 2. Methods

### 2.1. Approach

This study employs a narrative inquiry approach to explore the change in military identity among Canadian Veterans with chronic pain [30,31,32]. Narrative inquiry is a useful methodology for this topic because it can ground analysis in the lived, living, and evolving experiences of Veterans by capturing the nuances of how personal and institutional stories intertwine and influence each other over time. Moreover, this methodology is grounded in the understanding that identity is not a static trait, but a dynamic construct continually shaped by experiences, memories, and contextual influences [33]. In the context of Veterans with chronic pain, narrative inquiry allowed for an in-depth exploration of how chronic pain—an experience marked by physical, emotional, psychological and social dimensions—can disrupt and reshape identity across multiple life stages, from military service, transition to civilian life, and in civilian life. The findings presented in this paper are based on the results of the first set of interviews. The second interviews will inform a separate theory-focused paper focusing on how chronic pain disrupts and reconstructs military identity.

### 2.2. Sample and Recruitment

Eligible participants were Canadian Armed Forces [CAF] Veterans with chronic pain over the age of 18. We excluded individuals currently serving in the military, individuals under 18 years, and Veterans of non-Canadian militaries. Three Veterans also served in the Royal Canadian Mounted Police [RCMP] after serving in the CAF. We did not exclude Veterans depending on the time they served in the military. Participants were recruited through the Chronic Pain Centre of Excellence for Canadian Veterans listserv [>1000 members], a non-profit organization funded by Veterans Affairs Canada dedicated to funding research that explores the challenges associated with chronic pain among Veterans.

### 2.3. Data Collection

Data collection was implemented in two sequential phases of in-depth, semi-structured interviews designed to capture each Veteran’s narrative comprehensively. The first set of interviews [August to December 2024] probed Veterans’ experiences across three key stages: military service, transition to civilian life, and civilian life. These interviews focused on the onset, causes, and management of chronic pain, as well as challenges encountered at each stage.

One researcher (UM) conducted all interviews, except for two interviews with French Veterans, which a second researcher (LL) conducted. For interviews with French Veterans, the second researcher (LL) transcribed the discussions into French and translated the transcripts into English while ensuring that linguistic nuances were preserved. The French interviewer used back-translation to ensure that English transcripts and the findings in this paper accurately reflected the original meaning and nuances expressed by Veterans.

The data from these interviews informed the creation of two-page biographical accounts for each participant, which were then used in the second set of interviews [January to March 2025]. The second interview began with Veterans defining the concept of military identity, followed by the researcher reciting their biographical accounts and a set of questions focused on what military identity means to them and how chronic pain affects it. Post-interview reflexive memos were written to document emergent reflections, concepts, ideas, and potential analytic directions following interviews. The findings of this study focus on the data collected during the first set of interviews. Findings from the second interviews are being developed into separate manuscripts on how chronic pain disrupts and reconstructs identity change and how having both chronic pain and mental illness impacts Veterans’ everyday lives as civilians.

### 2.4. Data Analysis

Data analysis was conducted through an iterative, collaborative process that integrated contributions from a Veteran partner to ensure the analysis was grounded in authentic, lived, and living experiences. With the research team, a Veteran partner reviewed an initial set of transcripts to develop an inductive analytic framework by identifying key concepts, differences, and ideas emerging from data. This led the research team to develop an analysis template that maps key challenges, experiences, and milestones across the phases of military service, transition, and civilian life for each Veteran’s transcript, which was then applied to analyze remaining transcripts. All transcripts were analyzed by at least two researchers and disagreements were resolved through discussion.

Leveraging the analysis template and map of challenges and experiences, the researchers developed two-page biographical accounts to summarize each Veteran’s story, which were recited to them in the second interviews. These biographical accounts were used to conduct a reflexive thematic analysis [34] to identify factors that differentiate the experiences and challenges Veterans faced across their life stages. Through discussions with the research team and Veteran partner, one important factor was the type of injury and how chronic pain starts in the military, which we collectively refer to in this paper as three pain trajectories. Although we considered whether the chronic pain trajectories aligned with social identity characteristics, no meaningful patterns were observed. The three pain trajectories provided a critical lens for understanding how chronic pain can disrupt and potentially reshape military identity across the three life stages for Veterans.

## 3. Findings

In total, 20 Veterans were interviewed. A complete socio-demographic breakdown is provided in Table 1.

### 3.1. The Three Chronic Pain Trajectories for Canadian Veterans

The three pain trajectories described in this section convey differences in how chronic pain can shape and potentially unravel a once-firm military identity and propel Veterans into forging a new sense of self that combines aspects of the military life they left and the civilian life they experience every day [Table 2—a more comprehensive table of differences between trajectories is available in the Supplementary File]. In the first trajectory, Veterans who experienced a traumatic injury that led to an immediate discharge were faced with an abrupt disruption in their identity. On the other hand, in the second trajectory, Veterans who stayed in the forces despite serious injuries had to navigate everyday identity conflicts—remaining “soldiers” in name while coping with chronic pain that increasingly limited their physical abilities to live up to the expectations of those around them. Finally, in the third trajectory, Veterans who initially dismissed the pain as routine ‘wear-and-tear’ face encountered a more insidious form of identity disruption: the slow, gradual discovery that everyday life as a service member had become challenging, closely associated with ambiguity from not having pain associated with an accident or injury and a sense of weakness from not being able to perform as expected.

Although the findings are presented as three discrete trajectories, Veterans’ lived and living experiences rarely unfold in such tidy compartments. Military careers are marked by a succession of physical and psychological stressors, and each injury incurred along the way may correspond to a distinct pathway. For example, a Veteran might accumulate a series of micro-injuries that gradually intensify into chronic pain [trajectory 3], but then experience a traumatic injury event that causes immediate discharge [trajectory 1]. Similarly, another Veteran may continue to serve despite a serious injury [trajectory 2], while simultaneously managing pain in other areas of the body not directly linked to their injury and therefore do not have a clear label [trajectory 3]. By signposting these points of intersection throughout the Findings chapter, we underscore both the heterogeneity and the interconnectedness of Veterans’ pain narratives.

### 3.2. Trajectory 1: Traumatic Injury -> Immediate Discharge

**Figure 2:**
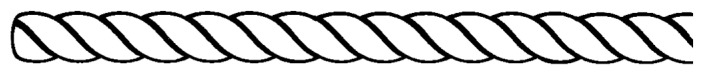
Rope depicting a traumatic injury that causes immediate discharge from military.

About one-third of the Veterans interviewed in this study experienced traumatic injuries that triggered a medical release from military service. The abruptness of the medical release is important because it reverberated through virtually every dimension of Veterans’ lives and created a premature end to their expectations of a full life in military uniform. The injury cut short their professional paths, jeopardized income stability, and—perhaps most profoundly ruptured the deep-seated identity they had constructed around a greater sense of purpose of serving in the military. This trajectory is visualized as a rope [Figure 2] that was cut without warning.

Although shifting to civilian life was difficult for all Veterans interviewed in this study, the group of Veterans who were released because of a traumatic injury or accident grappled with a particularly jarring transition from the need to navigate their traumatic injury and abrupt identity disruption at the same time. Their capacity to prepare psychologically or financially was minimal because the routines and camaraderie of military life vanished almost overnight, leaving at least some of them feeling both disoriented and betrayed by an institution that, in their view at the time of their release, offered neither the recognition nor the resources they desperately needed and hoped for. As a result, the Veterans’ self-concept as disciplined, purposeful service members was fundamentally shaken because of their injury—a reality that shaped their post-military careers and their broader sense of belonging and self-worth in the years of civilian life that followed.

A Veteran from the Army found himself abruptly released following a vehicle accident. Although he received two surgeries to address knee injuries while in the military, he was forced to leave just short of the 10-year threshold he needed to reach for a pension at that time in the military because of a sudden policy change. Once he left, he did not know what to do or where to go. The absence of financial security, combined with lingering chronic pain that kept getting worse, left him especially vulnerable as he fought [and continues to fight] for basic assistance and benefits for decades:

*“they screw up our calculations or don’t give us something we were supposed to get. You know, it’s constant with these guys…Yeah, so the government tends to drag their heels on that a lot, and it was never fun seeing our prime minister tell us that we’re asking for too much or more than they can give. That was a load of crap” [7257-Army]*.

A Veteran from the Air Force sustained a career-altering back injury while loading a missile, which ultimately led to medical release. Even though the injury took time to diagnose fully, his final moments of military life were unceremonious and exacerbated by constant harassment from his superiors who questioned his role and identity as a service member:

*“I wasn’t treated fairly…I think when I left, it was I felt like I was leaving with a bad mark to my name… he [supervisor] would berate me, belittle me, threaten my career, saying that ‘I’m going to make sure you’re released and you’re going to get nothing when you go’” [6009-Air Force]*.

The confluence of physical pain and a toxic workplace prompted him to embrace the transition to civilian life out of frustration, disillusionment, and a desire to escape an environment he no longer strongly identified with.

*“There was no real transition leaving the military to becoming a civilian. I was pretty much left to figure it out myself…I would let me hair grow longer, I wouldn’t iron my clothes, shine my boots… I just had an attitude” [6009-Air Force]*.

Fearing the potential consequences, a Veteran from the Navy did not report to the Medical Inspection Room [MIR] because of considerable pain caused by a slip during a training exercise. He believed that he would be dismissed and he would suffer ramifications with this team, including being perceived as weak for even going to the MIR. But eventually, the pain became so gruesome that he had to submit a request for voluntary release. However, without an MIR record, he remained ineligible for formal compensation for years after he left service, which put him in a precarious and frustrated state of mind. No compensation, military training not translating to the civilian world, and the need to provide caregiving to a spouse with multiple chronic conditions compelled him to juggle multiple jobs:

*“my military service did not transition into a civilian job…there was no way I was going to get straight out of the military and walk into another job” [7149-Navy]*.

The reality of having to juggle multiple jobs just to get by led to a situation where he so desperately wanted the VA to see the amount of pain he was in without any financial or psychological support:

*“I think it was just a kind of a swan song [at the VA], just to say goodbye, because I’m going to do something real stupid” [7149-Navy]*.

However, other Veterans proactively tried to mitigate some of the stressors associated with the dramatic change of being medically released after an injury. Two Army Veterans both attempted to mitigate the abrupt transition due to their traumatic injuries by proactively seeking civilian employment and establishing exit strategies on their own terms. However, the stress of juggling family responsibilities, numerous medical appointments, and uncertain military benefits reinforced a sense of suddenness and precariousness:

*“Sitting in a bureaucratic process waiting for your life to be adjudicated as having worth or not is fucking painful” [3528-Army]*.

Though the release was somewhat “planned” for this Veteran, it was still too sudden to provide the closure they needed to fully mitigate the identity disruption they faced:

*“this total distrust of the medical system and a further distrust of the administrative system like all piles in and so you know depression … regret of everything you’ve done thus far, you know, regretting getting hurt, regretting not taking better care of yourself, or regretting not making more of your career, and then massive amounts of anxiety for fear of the future” [3528-Army]*.

The experiences of this group of Veterans underscore how a single incident can have the potential to catapult service members into a disorienting separation from the military that they had hoped to call home for their entire lives. Veterans’ stories highlight the fragile nexus between physical trauma, bureaucratic systems, and psychological well-being. Often lacking sufficient documentation or institutional acknowledgment, Veterans departed service with unresolved grievances, a precarious financial footing, and an acute sense of unfinished business that they piece together through civilian occupations while navigating the chaos of civilian life with chronic pain that affects who they are and how they live. This abrupt severance from military life engendered unexpected challenges in reconciling their military identities with the realities of civilian life.

### 3.3. Trajectory 2: Misdiagnosed or Non-Traumatic Injury -> Delayed Discharge

**Figure 3:**
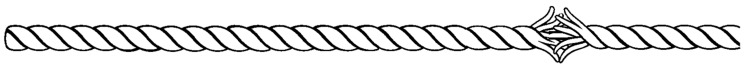
Rope depicting Veterans living with the effects of an injury in military life.

About two-thirds of Veterans sustained injuries that did not result in their immediate discharge because their injuries were misdiagnosed, underdiagnosed, or missed entirely. Whether caused by a single incident or a series of smaller injuries, these injuries lingered in the background of their careers and reframed the day-to-day of military life. This trajectory is visualized with a rope [Figure 3] remaining intact because they stay in military service, but fraying under pressure with time as chronic pain settles and gets worse in military service.

Veterans continued to wear the uniform—sometimes for years or even decades—yet they did so with bodies that gradually fell short of the military’s core ethos of peak fitness, constant readiness, and a stoic disregard for pain. This state of ‘staying in while hurting’ created the conditions for Veterans to engage in a unique, protracted negotiation of their military identity for the remainder of their career: on one hand, Veterans remained committed to the camaraderie, purpose, and prestige of serving; on the other, recurring pain and patchwork treatments eroded their confidence that they could fulfil the high standards demanded by both superiors and their own internalized sense of what it meant to be in the military. Over time, this internal dissonance often intensified frustration, isolation, and uncertainty about how they might eventually transition to civilian life.

A Military Police Veteran endured multiple injuries without prompt or effective treatment. His first injury from a rappelling accident was overlooked completely by his physicians, and only painkillers were offered for a subsequent vehicle accident. It was not until a third injury that led to a severe limb infection from fixing a valve in a flooded area that the military initiated the permanent medical release process. However, even this injury was initially misdiagnosed, leading to discussions of amputation, which were quickly corrected when treatment worked. Upon release, he found himself without the medical care or support needed, having lost the team of military physicians and therapists who had, however minimally, managed his pain over the years.

*“I guess you can in basic training… breaking you down, building you back up again to be military, but when they release you back into the civilian world, they don’t counter that, right? They don’t do anything to untrain you. From being institutionalized in the military, it’s just there’s the door, go use it. And then you’re out on the street. And then you’re without a doctor, without, you know, without anything, it’s this, there you go. There’s the door” [3735-Army]*.

Similarly, an Army and Air Force Veteran learned only after leaving service that his most severe fracture—a neck injury from a vehicle accident early in his 30-year career—went undetected for decades. But by the time he received complete imaging in his neck, he was diagnosed with a condition that led to a stroke while waiting for surgery that derailed his hope to serve in the military into his 60s. Had he only received complete imaging of his back and neck, maybe his life would have been different.

*“It was a real hard pill for me to swallow. My vision for my time in uniform was to stay in until I was 60. I had actually signed a contract CRA 60 to I had elected to stay in until age 60, because I love the work. I love the opportunities that the work gave me. You know, all my brothers and sisters that I grew up with were in uniform, you know, you know, the military was my family, you know. And when all of that stopped in [year of release], it was really difficult. I was not a happy person, you know, yeah, not happy at all” [6150-Army/Air Force]*.

He described how the “invisible” nature of a neck injury, because it was located behind his head out of his view, coupled with his desire to remain operational, contributed to multiple redeployments and tours, even though he was in immense pain throughout 30 years of service. His situation forced him to reconcile what he describes as a “failure” stemming from an enduring personal commitment to military service juxtaposed with the painful realization that his body’s deterioration was far more advanced than he or the institution recognized.

*“Throughout my entire training, my 30 plus years in uniform, and all the training that went with it, we were never taught what failure looks like. We were never taught how to fail …and how you accept that, how you rationalize that, how you understand that [failure]?” [6150-Army/Air Force]*.

A Military Police Veteran ignored early warnings from military physicians to slow down because he believed displaying ‘weakness’ would jeopardize his career progress. This decision gradually exacerbated his back issues. It was not an injury or accident, but a leave of absence taken to be with his fiancée, who had stage 4 cancer, that made him realize the “fragility of life.” This prompted him to shift attention from serving in the military to focusing on his health and well-being:

*“My focus in life had totally changed because of that two-year caregiving period and watching the fragility of life…And when I went back to work and walked in and looked at my boss and put in retirement papers on his desk and said, in six months time, I’m going be a memory. I said, I can’t do this anymore. It’s a young man’s game. Let the young fellas pick up the torch and run with it, because I’ve given my time, both mentally and physically, and I need to start living for me.” [8775-Military Police]*.

There is a central thread across these stories: continuing to serve with an injury positions Veterans in a space of enduring tension between their commitment to the military and the reality of a body in pain. Although remaining in uniform gave them a lasting sense of belonging and pride, the unrelenting discomfort—and often the lack of sustained, effective treatment—gradually eroded their self-confidence. Many described feeling they had to prove they were still capable, sometimes by downplaying their suffering or refusing to seek care to prevent a perception that they were unfit for duty.

*“I grew up with this in the back of my mind that if I, you know, sought out treatment for anything, I was weak. I wasn’t as strong as my brothers and sisters in uniform and so … [I] suffered from severe, chronic pain, fatigue and exhaustion every day. And I did all those things, and nobody would have known that there was anything wrong with me, you know, because I wouldn’t let them, I wouldn’t let them” [6150-Army/Air Force]*.

*“And military people don’t bitch and complain, because I was taught that pain is nature’s way of telling you you’re still alive. Don’t argue with it. Suck It up. Get on and capture the objective. That’s what the infantry does, right? So, you just do that. You self medicate out the wazoo and carry on” [2791-Army]*.

*“Yeah, pop some Tylenol and carry on. That’s the problem with, you know, being in the military, it’s suck it up and get back to work and, you know, and, and, of course, unfortunately, being a female, you know, you can’t show any weakness, because then the sharks gather in the water. So, and especially as a female leader, there’s even that much more pressure on you to perform at a higher level than your male counterpart” [1652-Army]*.

Unlike those whose injury led to an abrupt release, Veterans in this second pain trajectory had more time to anticipate their departure. But the protracted release process did not necessarily translate into a smoother civilian transition. The drawn-out nature of their final years of service engendered a kind of ‘limbo,’ where they continued to shoulder military responsibilities while navigating increasingly limited physical ability and a lack of institutional encouragement to do so. This was akin to an extended farewell, which might have dulled the shock of leaving uniformed life. Yet, it also amplified, in a sense, the sorrow, frustration, or quiet resignation that accompanied release. Ultimately, the experiences of this second group underscore the complexity of sustaining a military identity amid persistent pain that must reconcile the ideals of physical endurance with the lived, living, and evolving realities of chronic pain.

### 3.4. Trajectory 3: Cumulative ‘Wear and Tear’ -> Gradual Discharge

**Figure 4:**
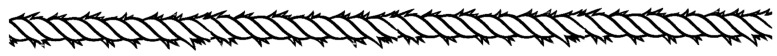
Rope depicting Veterans navigating regular wear and tear.

About one-third of Veterans developed chronic pain through a slow accumulation of micro-injuries, repetitive physical tasks, and sustained psychological stressors rather than a single or series of traumatic events or accidents. While this pattern of ‘wear and tear’ can appear less dramatic than a traumatic injury, Veterans’ accounts reveal that its impact on self-perception as a military service member, long-term health, and career trajectories can be equally significant—often more insidious as the gradual onset was associated with ambiguity around diagnosis and care. This trajectory is visualized as a rope [Figure 4] that is not broken but has thinned and frayed slowly as chronic pain settles and increasingly affects their ability to perform their job until there is nothing left to grip, which then causes them to leave military service.

For Veterans in this group, the conventional military culture of ‘pushing through’ coupled with limited recognition of hidden or less overt injuries meant that warning signs of chronic pain regularly went unnoticed or were dismissed as temporary aches that were expected in the military. This left Veterans to ‘soldier on’ until repeated physical strain morphed into chronic, life-altering pain.

An Air Force Veteran endured decades of lifting heavy equipment on tours but sought only minimal relief throughout his service when back pain flared, which involved muscle relaxants and/or bed rest.

*“And then I spent another day in bed, in the tent, because I didn’t want to take a hospital bed and so and then I went back to work again, doing everything again, trying to using painkillers. The military seem to like to patch you up with muscle relaxants and opioids” [8173-Air Force]*.

It was not until near the end of his service that more comprehensive diagnostic imaging revealed severe lumbar disc disease. Faced with the option of surgery, he opted instead for rehabilitation because of a fear of medical procedures and the expectation to return to duty as quickly as possible.

Similarly, another Air Force Veteran experienced progressive lower back, hip, and knee pain while dealing with sexual misconduct and limited medical support. The protracted nature of coping, akin to being a service member and reflective of the values instilled by military culture, left her feeling that she had spent years merely enduring pain rather than proactively addressing its root causes.

*“[There was a moment that] broke the glass and it was almost instantaneous where everything that had maybe been a dull roar before was now screaming the pain…I think of it as being a slow progression, and when I stopped to realize that, you know, all this stuff is real, it was immediate. It became, you know, like screaming pain versus the dull roar that I was trying to ignore” [7864-Air Force]*.

She resonated with removing her uniform and being part of a transition squad before her official release date, as it allowed her to begin ‘letting go’ of a military identity that had, in her view, become increasingly incompatible with her physical and emotional well-being.

*“It [transition squad] took me out of my environment…so that I could slow down and catch up with what is going on in my body…it gave me the chance to understand what was going on, and that’s where I was diagnosed with high blood pressure and some other things…[but] it was really hard that I took a big hit with that mentally, because I’ve always been active my entire life to suddenly being so disabled, it was really hard” [7864-Air Force]*.

Even among those who could point to a moderate incident, the overarching experience remained one of incremental deterioration. An Air Force Veteran sustained a back injury loading a missile, but he also grappled with persistent knee and foot problems that he linked to the equipment and footwear he used. Initially treated as muscular issues, these problems received only superficial care through physiotherapy, massage, and chiropractic sessions, and to him, it felt like re-injuring constantly:

*“And it was like I was reinjuring myself every, you know, not every time, but periodically, just reinjuring the neck and back. And it just never, never really improved” [6009-Air Force]*.

The underlying structural damage became apparent only after two years of minimal improvement, which prompted eventual imaging by a specialist. This delayed recognition highlights the subtlety of wear-and-tear conditions: what appears to be a manageable muscle strain can, in reality, mask deeper spinal or joint pathology, potentially leading to imaging later than when it would have provided the most relief and clarity. For Veterans in this group, the extended period of ambiguity around the cause of their pain amplified frustration and left them feeling less certain about their body’s reliability, representing a psychological shift in military identity. This group of Veterans often struggled with the perception, both their own and others’, that they were not “genuinely injured.” Without a clear, single precipitating event, their chronic pain was sometimes trivialized or deemed an inevitable side effect of military life.

*“We weren’t trained to worry about our feelings…you know, it was mission first. That was it, you know, mission before self. And it was just sort of drilled into you. You were just, it was physical. You were expected to be hurt. You were expected to freaking have bumps and bruises, and that’s just the way it was. And especially in the combat arms, if you can’t perform on the physical aspects, man, you better be prepared for it, because you’re definitely going to get rode hard and freaking, you won’t last if you can’t perform physically” [4119-Army]*.

Veterans in this group frequently underwent protracted, complex transitions out of uniform. The intangible, incremental emergence of chronic conditions blurred the demarcations between a ‘healthy soldier’ and an ‘injured soldier,’ leaving them uncertain about when or if they should seek release. Indeed, the point of separation often arrived only when the cumulative impact of wear and tear had become unmistakably debilitating and unignorable—or when Veterans, exhausted by sporadic or insufficient care, chose to leave rather than endure another cycle of inattention or partial solutions. In that sense, while their exit might seem more gradual compared to Veterans in the previous two trajectories, it remained marked by a sense of regret that their declining physical condition did not receive earlier institutional attention or more decisive treatment.

## 4. Discussion

The three pain trajectories discussed in this paper show a range of experiences from abrupt, destabilizing discharge to long, drawn-out processes of service riddled with challenges that question their physical capabilities because of chronic pain. Despite their differences, the common denominator of the three trajectories is the deep intertwining of chronic pain and identity. Each Veteran confronted the unsettling realization that physical debilitation associated with chronic pain undermined the core of what it means to be a Service Member. What emerges from this kind of identity dissonance is a pressing need for more responsive, individualized, and Veteran-centred services that appreciate and reflect the variety of paths by which injuries manifest. On the one hand, Veterans released rapidly might require immediate and thorough support so they can transition without feeling abandoned or invalidated. On the other hand, Veterans who remain in uniform while having chronic pain may need more flexible, proactive care while in service to ensure that both their physical and emotional well-being are prioritized and that identity dissonance does not lead to feelings of disillusionment. Finally, Veterans with gradual wear-and-tear highlight a potential opportunity and responsibility to detect, track, and address cumulative strains before they become chronic pain that then creates lingering, lifelong effects on Veterans’ lives and identities.

Although the findings are framed as three distinct chronic pain trajectories, Veterans’ experiences rarely aligned neatly within one trajectory. Many Veterans might carry multiple injuries and layered experiences of pain and their stories often straddled trajectories. For instance, a Veteran who had endured years of wear and tear might later sustain a traumatic injury that forced an abrupt discharge, while another might remain in uniform for decades despite one serious injury, only to accumulate other invisible injuries. In this sense, the trajectories are best understood as overlapping strands rather than mutually exclusive routes, where the interplay of physical injuries, institutional responses, and personal identities continually reshaped the course of Veterans’ lives.

Across these overlapping trajectories, a shared thread was the disruption of military identity. Regardless of whether the pain came suddenly from a traumatic injury, slowly from micro-strains, or insidiously through missed diagnoses, Veterans confronted the painful realization that they could no longer live up to the military’s ethos of strength, readiness, and resilience. For some, this disruption came abruptly, as when a traumatic event abruptly severed their careers and forced them into civilian life unprepared. For others, the process unfolded more gradually, as they remained in uniform but with bodies increasingly unable to keep pace with expectations. Even those who described years of pushing through wear and tear eventually reached a breaking point where the “soldier” they believed themselves to be no longer matched the reality of chronic pain.

Moreover, across all trajectories, chronic pain became tightly intertwined with emotional distress, experiences of institutional invalidation or mistrust, and ongoing struggles to reconcile military values with diminished physical capacity. Veterans in each trajectory described feeling a sense of unresolved grievance, whether from abrupt or delayed release, inadequate medical recognition, or the invisibility of cumulative wear-and-tear injuries. Taken together, these patterns underscore how chronic pain destabilizes military identity and complicates transitions into civilian life.

### 4.1. Developing Veteran-Centred Systems and Services Using Pain Trajectories

Veterans who experienced the first chronic pain trajectory underwent rapid, life-altering changes involving an abrupt severing of their military ties. The shock of immediate post-injury discharge forced them to confront not only declining physical functioning but also threats to their financial security and profound disruptions to their sense of self. This transition was particularly traumatic for Veterans who felt abandoned by an institution they had committed to with “unlimited liability” and no formal labour protections [35]. Unlike first responders, whose occupational health and safety standards and benefits are explicitly negotiated, safeguarded by unions, and enshrined clearly in legal frameworks such as Ontario’s Occupational Health and Safety Act, CAF members lack comparable legal protections or union representation. Without such safety nets, Veterans find themselves entirely dependent on VAC benefits legislation, subject to parliamentary discretion and potential policy volatility [36].

The unique concept of unlimited liability shapes military culture by setting an intense, pervasive tone that influences how military personnel act, behave, and engage, including their interactions with healthcare. Unlike first responders, who can legally refuse to undertake certain tasks if they compromise health and safety, military personnel operate under strict directives, where refusal to comply can result in severe consequences, including incarceration in Canada [37]. Such institutional dynamics engrain a culture of mission-first orientation and prioritization of group welfare above personal health and safety [38]. This cultural ethos does not end with active service; instead, it becomes an enduring part of Veterans’ identities, persisting into civilian life and complicating their navigation through post-service healthcare systems [38]. Veterans continue to internalize this ethos of self-sacrifice, which may inhibit their willingness or ability to advocate for their own health and potentially delay recognition and treatment of chronic pain and other service-related health conditions [38].

Veterans within the second chronic pain trajectory often contended with injuries overlooked or minimized over long periods, allowing continued military service at the expense of their long-term health. This prolonged tension, arising from the contradiction between declining physical capacities and ongoing service commitment, frequently amplified Veterans’ frustration with military healthcare structures, which some perceived as fragmented and inadequate. As injuries accumulated without adequate intervention, distrust in institutional accountability deepened [39]. This suggests a critical need for early detection and intervention protocols similar to initiatives such as screening programs developed by the United States Department of Veterans Affairs [40,41]. Implementing analogous preventive strategies in Canada could help address pain conditions earlier, thereby fostering trust in military healthcare and alleviating prolonged periods of uncertainty and frustration. Moreover, coupling early detection initiatives with education about pain science and the biopsychosocial dimensions of chronic pain—including the role of military identity—could enable military Veterans to articulate their healthcare needs more effectively during and after military service [42].

In the third trajectory, Veterans described a gradual, cumulative onset of pain through repetitive strain and chronic stress rather than discrete traumatic incidents. These Veterans emphasized even greater ambiguity, as neither the military nor the Veterans themselves clearly recognized when routine discomfort transformed into debilitating chronic conditions. Without identifiable injury events, Veterans in this group often found institutional acknowledgment elusive, leaving them with diminished legitimacy regarding their pain experiences [43]. This perceived illegitimacy, coupled with the absence of clearly documented medical histories, complicated their access to appropriate support systems and exacerbated feelings of institutional abandonment. Addressing these issues effectively requires embedding routine preventive healthcare measures and regular screenings specifically designed to identify micro-injuries and early symptoms of chronic conditions [44]. Equally important is educating service members about recognizing and articulating subtle pain symptoms early, fostering a proactive rather than reactive healthcare culture.

Furthermore, the concept of unlimited liability not only impacts Veterans’ immediate healthcare outcomes but also contributes significantly to the complexity of how they navigate their identity in the long-term. Unlike other marginalized or structurally disadvantaged communities, Veterans experience a distinct form of institutionalized restriction. Military training explicitly dismantles civilian identities, rebuilding service members within a rigid hierarchical structure defined by directives, compliance, and sacrifice. When these Veterans transition back into civilian society, especially with physical or psychological injuries, they confront additional, profound complexities beyond those of typical civilian life. The identity disruptions resulting from institutionalized military conditioning and subsequent re-entry into civilian society complicate Veterans’ efforts to seek and accept help, articulate their healthcare needs, and adjust to civilian support structures. What is potentially missing in this synthesis is how being in a strong, masculine military environment affects these chronic pain trajectories. We were unable to discern this aspect because of the low number of women in our sample, and this will be explored in a second paper on how chronic pain changes military identity from military service to civilian life.

For many women, becoming a Veteran entails negotiating a military identity built on hypermasculine norms and unequal power relations. Women’s stories from past research show how intersectional oppressions [sexism, racism, rank dynamics] and institutional betrayal complicate whether they feel seen as Service Members at all [45]. How invisible injuries during early reintegration make them compare unfavorably to male peers and feel dismissed in gender-insensitive systems [46]. The gendered hierarchy of “injury capital” pushes some to legitimize pain or distance from a disability label to avoid stigma [47]. Across three common chronic-pain trajectories discussed in this paper, these gender dynamics potentially play out differently:

1. After immediate discharge for traumatic injury, chronic pain can create the conditions for identity loss and contest the legitimacy of women whose sacrifice is constantly doubted.
2. With injuries that do not trigger immediate discharge, women often stay in uniform, mask pain to meet “toughness” expectations, and later navigate a fractured self.
3. With cumulative wear and tear, pain is often the least credible, amplifying disbelief, self-doubt, and invisibility.

### 4.2. Strengths and Limitations of This Study

A notable strength of this study was its nuanced exploration of the lived and living experiences of Canadian Veterans navigating chronic pain in civilian life. Including 20 Veterans from the Army and Air Force branches enriched the depth and range of insights generated. Furthermore, the intentional representation across diverse demographic and social identity factors—including different ages, ranks, military roles, lengths of service, and time elapsed since discharge—strengthens the transferability of findings and enhances understanding of how chronic pain intersects uniquely with Veteran identities. The narrative approach provides a poignant and powerful insight into how chronic pain affects identity, self-perception, and daily functioning.

However, interviewing Veterans who voluntarily responded to a call through the listserv of the Chronic Pain Centre of Excellence for Canadian Veterans introduces potential selection bias. Participants in this study were Veterans already connected to formal support networks, familiar with navigating online communication platforms, and currently receiving Veterans’ benefits. Focusing on Veterans who were already connected provided rich information as they were likely more comfortable with sharing their experiences, challenges, and stories. However, the experiences articulated in this paper might not capture the nuanced realities of Veterans with limited digital literacy, who face barriers accessing online resources, or remain disconnected from formal support systems, including those without VAC benefits. The absence of these narratives is particularly critical, as such Veterans may face heightened risks of identity disruption, isolation, or exacerbated chronic pain trajectories, perspectives that could alter or enrich the findings presented in this paper.

Finally, the work presented here was based on the first of two sets of interviews, which were deliberately designed to capture Veterans’ stories of injury, chronic pain and transition from military to civilian life without explicitly probing on identity. The second interviews focused on military identity and its relationship to chronic pain. While these later interviews likely deepened the understanding gained from the first interviews by adding new layers of meaning, the analyses presented in this paper stand on their own and reflect the intended design of the study.

## 5. Conclusions

This study highlights how chronic pain profoundly disrupts military identity across three distinct, overlapping trajectories. Whether through abrupt discharge, prolonged struggles with undetected conditions, or gradual wear and tear, Veterans consistently faced tensions between military ideals of stoicism and the lived and living realities of chronic pain. These experiences highlight the need for Veteran-centred approaches that acknowledge the diversity of pain trajectories, offer proactive and flexible care throughout service, and ensure responsive support during transition. Addressing these challenges is critical to safeguarding both the health and identity of Veterans navigating life with chronic pain during and after military service.

## Figures and Tables

**Table 1:** Socio-demographic breakdown for Veterans.

Characteristic		n [%]
Gender	Man	15 [75.0]
	Woman	5 [25.0]
Age	Mean	57.16 years
	Median	56 years
	40–49	3 [15.0]
	50–59	9 [45.0]
	60+	8 [40.0]
Race/Ethnicity	White	17 [85.0]
	Non-White	3 [15.0]
	French	2 [10.0]
Province	Alberta	6 [30.0]
	British Columbia	2 [10.0]
	Nova Scotia	2 [10.0]
	Ontario	6 [30.0]
	Quebec	3 [15.0]
	Saskatchewan	1 [5.0]
Rurality	Urban	9 [45.0]
	Suburban	4 [20.0]
	Rural	7 [35.0]
Highest Education	High school	6 [30.0]
	College	6 [30.0]
	University	5 [25.0]
	Graduate	3 [15.0]
Years of Service	Mean	24.08
	Median	24
	0–9	2 [10.0]
	10–19	4 [20.0]
	20–29	7 [35.0]
	30+	7 [35.0]
Year Released	Median	2016
	Mode	2019
	≤1999	3 [15.0]
	2000–2009	3 [15.0]
	2010–2019	12 [60.0]
	≥2020	2 [10.0]
Military Units *	Army	15 [75.0]
	Air Force	5 [25.0]
	Navy	3 [15.0]
Military Rank	Corporal	5 [25.0]
	Sargent	7 [35.0]
	Master Warrant Officer	1 [5.0]
	Captain	1 [5.0]
	Major	5 [25.0]
	Colonel	1 [5.0]
Chronic Pain Location Based on Diagnoses Self-Reported by Veterans	Back	19 [95.0]
	Knees/Legs	13 [65.0]
	Ankles/feet	7 [35.0]
	Hips	6 [30.0]
	Neck	5 [25.0]
	Elbows/Arm	5 [25.0]
	Wrist/Fingers	3 [15.0]
	Shoulder	2 [10.0]
	Jaw	1 [5.0]

* Three participants served in multiple military units.

**Table 2:** Three Chronic Pain Trajectories.

Traumatic Injury -> Immediate Discharge 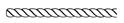	Misdiagnosed/Non-Traumatic Injury -> Delayed Discharge 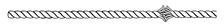	Cumulative ‘Wear and Tear’ -> Gradual Discharge 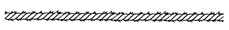
Veterans in this group experienced abrupt identity disruption when sudden injuries led to early medical release. Their pain was compounded by financial and occupational vulnerabilities, bureaucratic hurdles in securing benefits, and a lingering sense of betrayal by the institution. The immediate severing of ties with military life left many struggling to adjust to civilian life with unresolved grievances.	These Veterans remained in uniform for years despite chronic pain from misdiagnosed or undertreated injuries. They described conflict between the military ethos of strength and their physical limitations, creating identity dissonance and prolonged uncertainty. The drawn-out transition often left them unprepared for discharge, carrying frustrations about missed diagnoses and insufficient institutional support.	For Veterans with slowly developing pain, the lack of a clear injury made their conditions less visible and harder to validate. They faced delayed diagnoses, piecemeal medical care, and cultural pressure to “push through” pain. This invisibility left many feeling alienated, with protracted transitions out of service marked by exhaustion, regret, and doubts about whether the institution truly acknowledged their suffering

## Data Availability

The original contributions presented in this study are included in the article/Supplementary Material. Further inquiries can be directed to the corresponding author.

## Supplementary Materials

The following supporting information can be downloaded at: https://www.mdpi.com/article/10.3390/healthcare13202655/s1, Table S1: Comprehensive Description of Three Pain Trajectories.
