# Supplementary material for: Tracing How the Emergence of Chronic Pain Affects Military Identity: A Narrative Inquiry of Pain Trajectories Among Canadian Veterans
Source: Healthcare (Basel). 2025 Oct 21;13(20):2655. doi: 10.3390/healthcare13202655 (PMC12564806; doi:10.3390/healthcare13202655)
Supplement: Supplementary file 1 [file healthcare-13-02655-s001.zip › healthcare-3810072-supplementary.pdf]

Supplementary File

Table S1: Comprehensive Description of Three Pain Trajectories.

	**Traumatic injury -> Immediate Discharge**	**Misdiagnosed/Non-Traumatic Injury -> Delayed Discharge**	**Cumulative 'wear and tear' -> Gradual Discharge**
	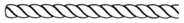	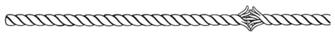	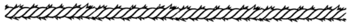
**Identity Changes**	**Identity Disruption due to an Abrupt End to Military Service:** The injuries that precipitated medical release severed a deep-rooted sense of purpose tied to service, leading to premature end to career aspirations and halting expected pensions or benefits. Veterans found themselves suddenly propelled into civilian life without the psychological or financial preparation they needed, complicating the transition. The shock of losing their military community and professional identity in an instant led to feelings of disorientation and unfinished business.	**Conflict Between Military Ethos and Physical Realities:** An essential part of military identity is projecting strength, endurance, and stoicism. Veterans in this group felt immense pressure to hide or downplay their symptoms in the military to avoid being perceived as weak or unfit for duty. Over time, the sharp contrast between the ideals of constant readiness and the unrelenting physical toll of their injuries intensified their sense of inadequacy and self-doubt.	**Military Culture of Stoicism and 'Pushing Through':** Veterans felt institutional and cultural pressure in the military to keep silent about nagging pains for fear of being perceived as weak or unfit. This ethos of silent endurance led some to rely on short-term fixes—such as muscle relaxants and bed rest—rather than seeking deeper medical intervention, perpetuating the cycle of injuries that went unrecognized or under-addressed for years, eventually leading chronic pain end of service or much after retiring.
**Emotional Dimensions**	**Interplay of Chronic Pain and Emotional Distress:** Persistent physical pain from traumatic injuries compounded emotional distress, especially when military policies or leadership casted doubt on the legitimacy of their conditions. Veterans confronted lingering pain while trying to secure benefits, compounding their stress and undermining their confidence in the military's medical and administrative systems. This inextricable link between chronic pain and emotional distress often shaped their ongoing	**Psychological Tension and Identity Dissonance:** Remaining in service with undiagnosed or undertreated pain generated a sense of limbo—trying to uphold the values and responsibilities of the military while privately bearing physical discomfort. The mismatch between who they were expected to be (i.e., healthy, ready combatants) and who they had become (i.e., wounded, uncertain) led many to grapple with deeper questions about self-worth, belonging, and the meaning of 'failure' in the military context.	**The Psychological Toll of 'Invisible' Injuries:** The absence of a clear, precipitating event sometimes led both peers and superiors to question the legitimacy of their pain. This left Veterans feeling invalidated and increasingly alienated. The lack of clear acknowledgment of 'wear and tear' also undermined Veterans' confidence in their own bodies and blurred the lines between being a healthy Service Member and one in slow decline.

	struggles to find meaning and stability in civilian life.		
**Institutional Experiences**	**Perceived Institutional Betrayal Because of Bureaucratic Hurdles:** Veterans felt betrayed by superiors who, in their view, dismissed or weaponized their injuries against them, leading to a deep-seated distrust in the institution they once served with pride. Policies and procedures, including unclear discharge processes and miscalculations of benefits, fostered frustration and resentment. Veterans described the slow pace of government agencies, administrative oversights, and conflicting information as tangible barriers to receiving much-needed recognition and support.	**Lingering Injuries from Extended Military Service:** Veterans in this group sustained physical injuries that were initially missed, minimized, or underdiagnosed. These Veterans remained in uniform—sometimes for decades—working through pain that eroded their trust in the institution's capacity to detect and treat injuries early on. While wearing the uniform gave them more time to serve, it also set them on a protracted path of chronic pain and slow-burning disenchantment.	**Delayed Diagnosis and Fragmented Medical Attention:** Veterans described receiving piecemeal care (physiotherapy, massages, and occasional imaging), which often targeted immediate symptoms rather than probing for underlying causes of pain. Many realized only late in their careers that their conditions were far more serious and might have been diagnosed earlier with more consistent or thorough clinical assessments.<br><br>**Gradual Onset of Chronic Pain:** The slow build-up of pain often made it difficult for them and their physicians to pinpoint a specific cause, fostering an environment where persistent aches were dismissed as part of the job rather than signals of looming chronic conditions.
**Transition Experiences**	**Financial and Occupational Vulnerabilities Post-Discharge:** The abrupt termination of military careers left many Veterans without stable income streams or the time to realign their skills with civilian job opportunities. Skill sets like those acquired through military electrician training did not translate readily to civilian certifications, adding to their economic and personal strain. Some found themselves juggling multiple low-paying jobs or forced to rely on family networks or precarious forms of income, heightening the urgency of securing official benefits and compensation.<br><br>**Lingering Sense of Unresolved Grievance:** While a few Veterans took steps to control their exit by seeking civilian employment or planning around	**Extended Farewell:** Though they were not discharged outright after the injury, having time did not guarantee an easier transition. The drawn-out years spent coping with pain and waiting for the inevitable discharge often turned into a prolonged emotional farewell rather than a clear, well-supported transition plan. Veterans described feeling unsupported by leaders and military bureaucracies that struggled to adapt to their evolving physical realities, leaving them still emotionally unprepared when discharge finally arrived.<br><br>**Unresolved Grievances:** Many left service with unresolved medical and administrative frustrations—sometimes discovering long-misdiagnosed fractures or overlooked conditions	**Protracted and Uncertain Transition Out of Uniform:** The gradual nature of these injuries meant that many served for years under ever-growing physical strain, often exiting service only when the cumulative toll became impossible to ignore. While this trajectory may appear less abrupt than a sudden medical release, it left Veterans with regrets and unresolved concerns about the delayed recognition of their condition. By the time they left service, many felt exhausted from years of insufficient care, profoundly impacting both their transition and their sense of whether the institution had truly looked out for their well-being.

	medical appointments, they still faced sudden policy shifts, uncertainty in benefits, and a lack of closure around leaving their military life. Long after discharge, participants carried unresolved grievances—about both the abrupt release and the institutional response—that continued to shape their perceptions of self-worth and overall well-being.	only after discharge. Lingering doubts about whether the military could have intervened sooner or provided better care created a profound sense of betrayal. Even when they knew departure was coming, many emerged from service feeling psychologically unmoored, financially uncertain, and questioning the institution they once trusted to safeguard their well-being.
